# Proposing a holistic research framework for university strategic alliances in sustainable entrepreneurship

**DOI:** 10.1016/j.heliyon.2023.e16087

**Published:** 2023-05-06

**Authors:** Dina Pereira, João Leitão, Tiago Oliveira, Dario Peirone

**Affiliations:** a University of Beira Interior, Faculty of Human and Social Sciences, NECE – Research Centre in Business Sciences, Estrada do Sineiro, 6200-001 Covilhã, Portugal; b Centre for Management Studies of Instituto Superior Técnico (CEG-IST), University of Lisbon, 1649-004 Lisboa, Portugal; c Research Centre in Business Sciences (NECE), University of Beira Interior, 6200-209 Covilhã, Portugal; d Instituto de Ciências Sociais (ICS), University of Lisbon, 1649-004 Lisboa, Portugal; e Dipartimento di Giurisprudenza, Università degli Studi di Torino, Campus “Luigi Einaudi” Lungo Dora Siena, 100, A10153 Torino, Italy

**Keywords:** University-university collaboration, Sustainable entrepreneurship, Sustainable development

## Abstract

This study presents a systematic literature review aimed at mapping the main areas of study on the relationship between higher education institutions' strategic alliances and sustainable entrepreneurship. To that end, it carried out three complementary analyses: topic mapping, co-citation, and overlay visualization, in order to provide a comprehensive picture of that relationship from 1994 to 2022. The empirical approach is based on a total sample of 207 articles published in the Web of Science database, which was screened in terms of title, abstract and keywords, and subject to a search protocol involving inclusion and exclusion criteria. Using VOSviewer software, a three-pronged approach is used to identify five topic clusters: (1) The impact of entrepreneurship on community sustainability and social innovation; (2) Strategic alliances for sustainable development, innovation, and performance; (3) Value creation through social entrepreneurship partnerships; (4) Challenges for knowledge-based sustainable cities; and (5) Collaboration between businesses and social enterprises; revealing the role of knowledge, co-creation, sustainable entrepreneurship, and social innovation as levers of sustainable development. As a result of this systematic literature review, a holistic research framework is proposed, positioning sustainable entrepreneurship as a priority target for strategic alliances in higher education institutions, with reference to the experience of implementing the European University concept. This framework helps to position joint cooperation and strategic alliances among the major stakeholders in knowledge-based economies, which frequently leads to knowledge-based development based on sustainable entrepreneurship.

## 1 Introduction

Over the last few years there has been ongoing debate about the dimension and extent of European Universities, with the development and fostering of cooperation among Higher Education Institutions (HEIs) in order to develop a true ‘European University’. This concept was highlighted in 2017 in Gothenburg at the European Council, encouraging the HEI cooperation network to foster opportunities for students to engage in multiple combined studies in several HEIs from various European Union (EU) countries [1]. This would lead to broader international competitiveness, not only for the HEIs involved, but also for the students, who could obtain their degree and enrol in an international mobility experience.

The concept of the ‘European University’ was designed in order to promote the values of a true unified Europe, with common goals, identity and values, as referred to in the Treaty of Lisbon, thus enhancing the quality and competitiveness of European HEIs [2]. Consequently, the EU's “Europe 2020” aimed for inclusive and sustainable growth, giving an important role to joint cooperation or strategic alliances between HEIs. This included the implementation of joint and international degrees and doctoral programmes, staff and student mobility, research consortia, start-up acceleration, technological entrepreneurship, and sustainable development education programmes, also establishing open innovation schemes with the business sector to enhance the competitiveness of the surrounding communities [3].

Following [4], strategic alliances are a vital means of achieving knowledge and technology transfer, across various organizational barriers, cutting across the countries where the partners are located. As stated in Ref. [5] strategic alliances as conscious agreements between several parties to exchange or share knowledge or resources, so that one or all of them can easily develop processes or products/services. Therefore, through a strategic alliance, partners' knowledge will be assimilated and transformed into the company's own resource [6]. According to the [7], this university-university collaboration is developed through joint research activities and technology transfer, leading to co-publications and patents, as well as stimulating mobility not only for students, but also for lecturers, non-teaching staff and researchers.

In addition, creating or developing goods and services through strategic alliances or collaborations should consider not only the economic, psychological and social, but also the environmental consequences [8]. This is also partly related to the strategies being designed by companies or countries, whereby green and digital transformations regarding sustainability are meant to be the drivers of sustainable growth [9]. If collaborations are formed in order to solve or improve societal and environmental problems, they will be linked to sustainable entrepreneurship [10]. This is in line with [11]'s vision, which advocates that cooperation relationships are the stepping stone for a societal shift towards sustainability.

Considering joint cooperation leading to strategic alliances, and sustainable entrepreneurship, several research papers have focused on University-Industry cooperation. However, few have studied University-University joint cooperation or HEI-HEI strategic alliances directed to sustainable entrepreneurship or linked to that subject in particular. Therefore, this systematic literature review (SLR) analyses the main studies on these types of joint cooperation, as well as listing the main themes in published work, identifying research gaps, proposing a holistic research framework, and indicating possibilities for future research.

In general, the current SLR aims to identify the main areas of study on the relationship between HEIs' strategic alliances and sustainable entrepreneurship. Specifically, it aims to design a framework for better understanding of how the main actors in the knowledge-based economy can ensure knowledge-based development, through joint cooperation and strategic alliances, taking as a benchmark the lessons originating in the innovative initiative of European University alliances.

According to the objectives of this study, the following research questions are formulated:

Question 1 (Q1). What are the main areas of study on the relationship between HEIs' strategic alliances and sustainable entrepreneurship?

Question 2 (Q2). How can joint cooperation and strategic alliances between the main actors of knowledge-based economies contribute to knowledge-based development and achievement of the Sustainable Development Goals (SDGs)?

Considering the results of the bibliometric analysis in the current SLR, five topic clusters are identified, namely: (1) Entrepreneurship's impact on community sustainability and social innovation; (2) Strategic alliances for sustainable development, innovation and performance; (3) Value creation through partnerships in social entrepreneurship; (4) Challenges for knowledge-based sustainable cities; and (5) Collaboration between businesses and social enterprises.

By integrating the five topic clusters, a holistic framework is provided for both policy-makers and scholars for future action and research endeavours, believing that the parts of something are intimately interconnected and explicable only by reference to the whole.

Based on the recent experience of implementing the European University idea, the findings of this bibliometric research suggest that sustainable entrepreneurship should be a priority for strategic alliances at HEIs.

In this line of reasoning, to pursue this priority, there is a need to integrate the five topic clusters, addressing University-Industry relations, University-University relations, sustainable entrepreneurship, the social impact of universities, and the importance of alternative funding mechanisms for social and environmental innovation activities, to foster universities’ strategic positioning, towards achieving the SDGs.

The present study originates in the set of activities included in the European project InnoUNITA (Innovation capacity building in UNITA), which is financed through the EIT Pilot Program HEI Initiative. This project aims to position HEIs in local economic ecosystems so that they can develop and support entrepreneurship in the territories where they are located. InnoUNITA is a part of the UNITA – *Universitas Motium*,^1^ which is a European University Alliance, embracing 6 national HEIs, in 5 EU countries, with more than 160 thousand students and 15 thousand members of staff.

The remainder of this study is structured as follows. First, a review of the theoretical background is provided. Second, the methodological design, procedures and data preparation for the SLR are presented. Third, using a three-pronged approach based on: topic mapping, co-citation and overlay visualization, the empirical results are revealed. Fourth, the results are discussed, including a new research framework to address university strategic alliances in sustainable entrepreneurship, based on a two-fold knowledge approach. Finally, the concluding remarks, limitations and future research avenues are presented.

## 2 Theoretical background

### 2.1 Joint cooperation leading to strategic alliances

Joint Cooperation is highlighted as leading to better products and services in companies. Parties cooperate to create strategies that will generate value and reach a common goal (despite the complexity and risk associated with all types of collaboration), sharing resources [12]. Cooperation is also seen as a joint search for a goal defined in a previous agreement, and leading to a contribution or reimbursement for all cooperating parties [13]. Adding to the previous, cooperation can be positioned as the pursuit of a common benefit through an alliance of mutual interest [14]. Nevertheless, research has traditionally criticised cooperation, mainly because of the competitive logic between parties, with cooperation being seen as a way to restrain competition [15]. Since cooperating with other firms means greater experience, this will lead to a broader range of collaborative efforts, refining routines, leading to greater versatility and allowing other types of cooperation [16], even with competitors.

It is also important to analyse “cooperation with competitors”, or coopetition, as stated by Ref. [14], where cooperation as well as competition among partners is seen as a strategic premise of an alliance. There is direct or indirect competition between the parties in the alliance, with potential future competition for resources (including human resources), knowledge or even the technology generated in the alliance. Coopetition gives the opportunity of benefiting from competition, leading to better performance [17]. This dichotomy between cooperation and competition can be the most advantageous connection between partners/competitors [18]. Despite the benefits of coopetition, such as spurring innovations, economic performance, sharing joint costs, risks, and expertise, achieving scale dimension, and pooling R&D activities, coopetition is not a risk-free strategy, as it can lead to opportunistic behaviours, conflicts of interest or a loss of flexibility [19,20].

Strategic alliances are defined by Ref. [4] as interfirm cooperation which uses knowledge, resources or physical or organizational structure. As advocated by Ref. [21] an enhanced strategic alliance risk is shared among the partners. This can even migrate to a co-creation basis, if the partnership is based on open business models, involving the customer as a co-creator of value [22]. For example, in the scope of the European University Alliance, several transnational open innovation competitions have been organized, functioning as open innovation labs, where companies propose challenges (real business problems) to be solved by researchers, scholars, entrepreneurs and public bodies. This type of initiative not only reinforces the need for research consortia integrating HEIs, Industry, Government and Citizens, but also contributes to increasing the number of start-ups to provide viable solutions oriented to SDGs. The strategic alliance is generally seen as a hybrid arrangement, where parties balance their joint purpose in market transitions in a horizontal relationship [23]. In the scope of the European University Alliance, it is worth mentioning the establishment of several horizontal agreements especially involving HEIs and entrepreneurial ecosystems in Europe, perfectly articulated with several branches of the European Institute of Technology (EIT), namely EIT Food and EIT Manufacturing, as well as the EIT HEI Initiative including the EcoAction initiative.^2^

HEIs' contribution to the development of their regions has been highly praised in several studies, mostly through technology transfer [24], but also knowledge transfer [25], transforming HEIs by capitalizing knowledge and becoming an entrepreneurial university, with economic development becoming as important as research or teaching [26]. This has become known as HEIs’ “third mission” [27].

HEIs’ “third mission” involves a series of relationships with industry and government (as stated before), as an important part of the “Triple Helix” [28]. As well as these types of interactions, HEIs engage in university-university or inter-university cooperation, helping them to improve their knowledge [29]. Moreover, the strategic alliances among HEIs are usually related to developing greater, more focused HEI scholar programmes, allowing them to have a more diverse and competitive approach [30]. According to the [7], cooperation between HEIs is based on close investigation, exchange programmes, or joint-research, which enables an exchange of information and science-making processes, increasing co-publications in international journals, the exchange of staff and co-participation in developing industrial property. The importance of HEI-HEI cooperation and collaboration is outlined by Ref. [31], giving both parties knowledge and different points of view on doing science, since these alliances are made with partners from different parts of the world. Regarding sustainable inter-university collaboration [32], state that this connection enhances interpersonal relationships, as well as strengthening professional development.

University-University cooperation is sometimes viewed as a complex and difficult engagement, since there are different visions of the partnership, and different views on the results, regarding their translation to industry or remaining only as research and publication [33]. The pressure and responsibility regarding inter-university cooperation is difficult to handle, leading to failure of the collaboration [34]. This collaboration/competition by HEIs can be enhancing as well as undermining, depending on the types of inter-organizational arrangement, or even if they are effective on an international, national, or regional basis [35].

Cooperation between companies and HEIs is perceived as of major importance in spurring competitiveness on a regional basis [36]. The development of a “Triple Helix” relationship between HEIs, Industry and Government can be interpreted as a starting point of a knowledge-based society [37]. Within this “Triple Helix”, HEIs are active participants in developing the regional economy, as well as the social part of society, establishing strong cooperation with (local) government and industry, although depending on the place where these alliances and cooperation are formed [28]. The lack of strong cooperation between the three major players (HEIs, Industry and Government) in the “Triple Helix” and society paved the way for the creation of a “Quadruple Helix” (HEIs, Industry, Governments and Citizens), as stated by Ref. [38]. This “Quadruple Helix” can be seen as human-centred cooperation between the actors, opening up knowledge and thus leading to the development of art-based innovation [39]. A fifth helix (identified as “the natural environment”) is proposed in Ref. [40], which also included a to create a “Quintuple Helix”, which includes the linkage between social interactions, promoting cooperation for knowledge, involving HEIs, Industry, Governments, Citizens and Sustainability, oriented to innovation and sustainable development.

### 2.2 Joint cooperation and strategic alliances for sustainable entrepreneurship

Due to the type of life we lead nowadays and the resulting damage to the environment, in 2015, 193 countries defined and adopted the SDGs, as a common agenda of 17 goals to be reached by 2030 [41]. With this in mind, it has been increasingly important to connect and find ways to link existing knowledge about sustainability and the environment through cooperation [11].

As argued by Ref. [42], cooperation, alliances or partnerships are vital for sustainable development, since they bring several stakeholders together to decide on the necessary conditions for creating a new product or service, in order to meet the criteria of sustainability. Conversely, innovation systems focus on diverse stakeholders, such as firms, HEIs, policy makers and consumers, and on links between those, namely R&D collaborations, knowledge transfer, flows of goods or user-producer joint cooperation schemes. These systems allow for common learning and knowledge generation, to assure possible modes for sustainability transformation [43].

Following the statement provided by Ref. [41], reaching the 17 SDGs is a transversal task, involving governments and companies, as well as engaging other kinds of stakeholders, which would lead in a broader sense to international cooperation. This need could increase governments’ intention to work with different stakeholders, creating multi-stakeholder partnerships in order to reach policy and institutional coherence, as well as data, monitoring and accountability, so that those SDGs can be achieved. It is also important to point out that for the [44], the 2030 Agenda for Sustainable Development requires a major increase in resources, as well as a massive collective effort. The necessary funding must come through governmental partnerships with the private sector, joining efforts in order to solve sustainable problems at a planetary level. That is shown by the [41] when stating that the basic principles of the SDGs (affirmed to be Planet, People, Prosperity, Pace and Partnership) lead to partnerships as an essential way to reach the goals. This was also an issue tackled by Ref. [45], stating that this connection between sustainable entrepreneurs, stakeholders and regulators leads the way in analysing strategies on sustainability and the environmental well-being of communities.

It is important to define the sustainable entrepreneur and sustainable entrepreneurship. Considering the conceptualization proposed by Ref. [46], the sustainable entrepreneur is the one who can balance economic health and social fairness, as well as environmental awareness, through entrepreneurial behaviour. In turn, in Ref. [47] sustainable entrepreneurship is defined as the procedure of analysing the market, trying to find the breaks concerning the environment and its sustainability, evaluating and exploiting them, and thus turning them into economic opportunities. Furthermore, in Ref. [48] is argued that sustainable entrepreneurship is linked to entrepreneurial opportunities related to the creation or discovery of products or services that will be good for the community environment. This is particularly relevant in the scope of the UNITA - European University Alliance, since all the research hubs are directly connected with the SDGs, including the circular economy, cultural heritage and energy efficiency. In addition, all research projects and start-ups require identification of the SDGs addressed in these innovative, entrepreneurial initiatives.

According to Ref. [49], this link between the public and private sectors is based on the principle that although governments have added value in their positioning with regard to the SDGs, there are also difficulties in terms of financing (as already stated) if governments act alone. Although these partnerships are the most comprehensive and fastest way to achieve the SDGs, other factors may limit achievement of the SDGs through partnerships. These include the difficulty of working together, or organizations’ vested interests, putting those interests before the achievement of goals.

## 3 Methodological design, procedures, and data preparation

An SLR is the best way to identify, evaluate and interpret the available data which has already been condensed and produced by other researchers, allowing a greater structure of a specific field of knowledge and leading to new forms of understanding and connection [50,51]. Following the rationale presented by Ref. [52], an SLR is meant to be more objective and transparent than traditional reviews, thus providing guidance for future researchers and relevant information on the issues they mean to research [53]. It is also important to mention that an SLR has a transparent procedure that can be replicated, allowing researchers to be more effective throughout the whole process, analysing all the factors of the research made by others [54].

Following [55], a triangulation approach is used to delve deeper into the SLR results, namely a three-pronged approach involving topic mapping, co-citation and overlay visualization. This procedure is justified by their complementarity and the fact that additional, profound inferences can be drawn from data, as well as increased validity by comparing various techniques. The use of SLR and bibliometric analysis techniques is critical to provide an accurate knowledge map of the topics in the current study [56].

**Fig. 1:**
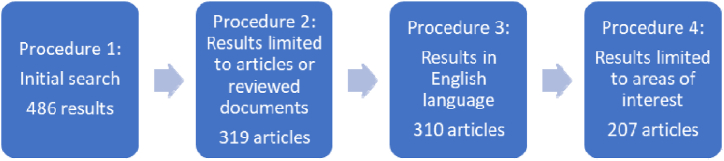
Research protocol procedures: Database building (Source: Own elaboration).

For this SLR, Clarivate's Web of Science (WoS) database was chosen to perform a search in April 2022. The database included data from 1994, as there are publications from that time until the moment of this retrieval. WoS was chosen as the database for this bibliometric study because it contains journals and articles on a wide range of subjects. According to Ref. [51], it is also the most used database for studies involving management and organizational issues (relevant for this SLR). Fig. 1 depicts the structure and all procedures of the research protocol for data preparation for use on the SLR.

A process of co-occurrences was used for database construction, as shown in procedure 1 of the analysis. Topic mapping reveals latent information in large bibliographic sets by comparing two probabilistic distributions and returning clusters of topics and their linkages in a graphical and visual mode [[57], [58], [59]]. To process the analysis, the following string was used: (sustainable) AND (entrepreneur*) AND ((joint cooperation) OR (strategic alliance) OR (partnership)). Searching for these terms returned 486 results. In procedure 2, we limited the search to “articles” or “reviewed documents”, eliminating “books”, “book chapters”, “conference reports” and “proceeding papers”, which retrieved 319 results. Applying procedure 3 filtered the results to those written in English, giving 310 results. Lastly, procedure 4 limited the results to the areas of interest for this SLR: Business, Management, Regional Urban Planning, Economics, Green Sustainable Science Technology, Social Sciences Interdisciplinary, Business Finance, Public Administration, and International Relations. After that, we read and analysed the titles, abstracts and keywords, taking a full analysis of the article, whenever necessary. This search returned a final sample of 207 articles. The inclusion and exclusion criteria used are presented in Table 1 below.

**Table 1:** Inclusion and exclusion criteria.

Inclusion Criteria	Exclusion Criteria
Present in Clarivate's Web of Science	Books, Book Chapters, Conference Reports, Proceeding Papers
Published in English	Non-English Publications
Article or Reviewed Article	
Until May 2022	
Areas of interest: Business, Management, Regional Urban Planning, Economics, Green Sustainable Science Technology, Social Sciences Interdisciplinary, Business Finance, Public Administration, and International Relations	

**Fig. 2:**
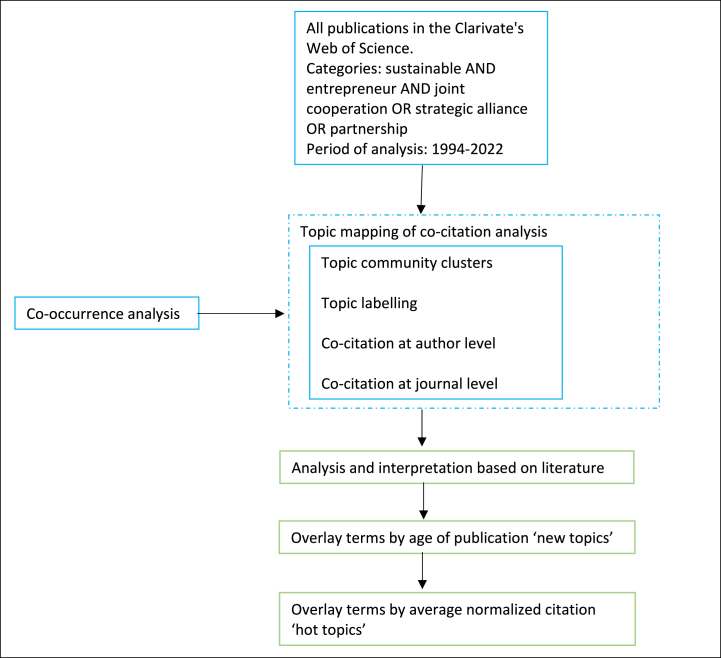
Research design (Source: own elaboration).

From this, co-occurrence networks were constructed, choosing the most relevant keywords to create the topic mapping. Fig. 2 shows the research design used.

Co-citation analysis was then used to perform overlay visualization techniques at the author and journal levels. This type of analysis highlights important latent relationships between authors and journals, translating them into visual outputs, such as co-citation clusters, and retrieving additional insights validating topic mapping [55]. This tool clusters related publications using Van Eck's clustering technique [60]. To perform the co-citation analysis, a minimum of 20 citations were used as the threshold, resulting in images that are clearer and less crowded than other work with 5, 10, or 15 citations.

The overlay visualization analysis was used to reveal the most recent trends in publications, as well as the hottest topics. This type of tool provides critical information about the research path and trends in a specific field.

The above-mentioned three-pronged approach is based on the previous work developed by Ref. [55], and was chosen for the complementarity it provides by synthesizing, inferring, and graphically presenting the newest and hottest topics in the publications under analysis.

## 4 Results and discussion

### 4.1 Descriptive analysis

**Fig. 3:**
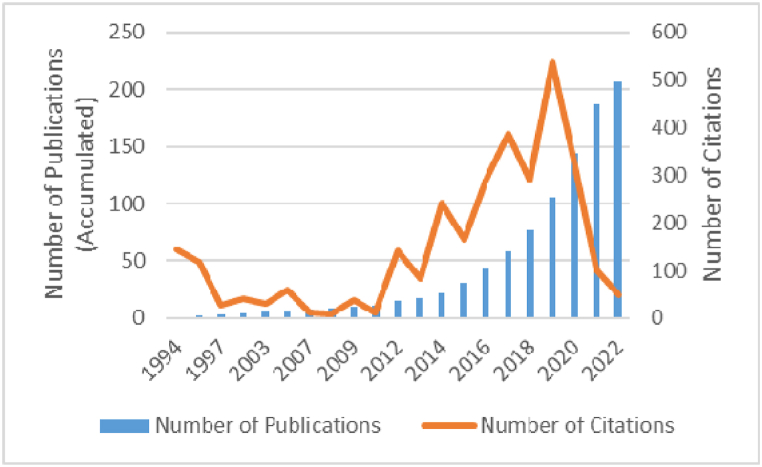
Number of publications and citations over time (Source: own elaboration).

A first descriptive analysis of the publications and publication years is presented in Fig. 3. This shows the increasing tendency of articles published, with several fluctuations on the timeline. The greatest increases are in the last two years (2020 and 2021), the latter having most, with a total of 43 published articles, which represents the increasing and current importance of the theme. It is also important to point out that the last year (2022) is only up to April. Regarding citations, despite fluctuations the importance of the topic is seen to have increased since 2019, with this year having the greatest number of citations (538).

**Fig. 4:**
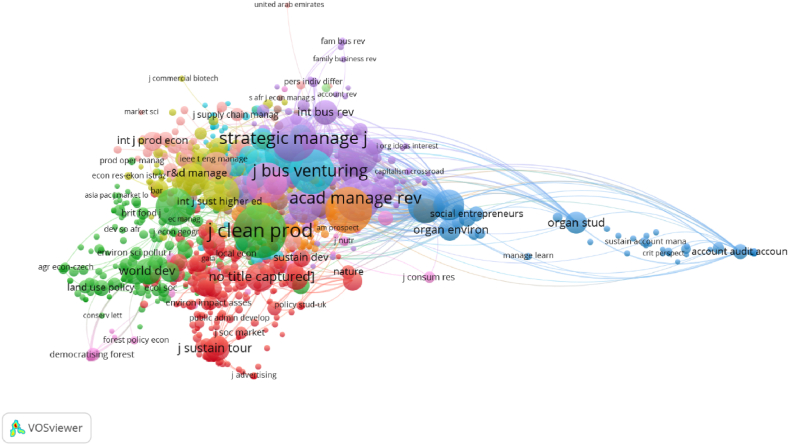
Co-cited Journals (Source: own elaboration using VOS viewer software).

Table 2 shows the journals with most publications regarding the selected articles. “Sustainability” is found to have the greater number of publications on this topic, with 29. Following this, the most prominent journals are “Journal of Cleaner Production”, “World Journal of Entrepreneurship Management and Sustainable Development”, “Journal of Sustainable Tourism”, “Business Strategy and the Environment”, “Entrepreneurship and Sustainability Issues”, “Baltic Journal of Economic Studies”, “International Journal of Sustainability in Higher Education”, Journal of Business Ethics”, and “Journal of Business Venturing”. Regarding the most co-cited journals in the scope of this SLR, “Journal of Cleaner Production”, “Journal of Business Venturing”, “Strategic Management Journal”, and “Journal of Business Ethics” have the highest number of co-citations (see Fig. 4).

**Table 2:** Journals with the highest number of publications.

Journal Name	Number of Publications
SUSTAINABILITY	29
JOURNAL OF CLEANER PRODUCTION	12
WORLD JOURNAL OF ENTREPRENEURSHIP MANAGEMENT AND SUSTAINABLE DEVELOPMENT	9
JOURNAL OF SUSTAINABLE TOURISM	8
BUSINESS STRATEGY AND THE ENVIRONMENT	6
ENTREPRENEURSHIP AND SUSTAINABILITY ISSUES	6
BALTIC JOURNAL OF ECONOMIC STUDIES	4
INTERNATIONAL JOURNAL OF SUSTAINABILITY IN HIGHER EDUCATION	4
JOURNAL OF BUSINESS ETHICS	4
JOURNAL OF BUSINESS VENTURING	4

The top ten articles with both citations and links are listed in Table 3 below. The top three articles in terms of citations are [61,62], and [63].

**Table 3:** Top cited publications.

Title of the Publications	Studies	Journal	Year	Citations	Links
‘Give me a laboratory and I will lower your carbon footprint!’ – Urban Laboratories and the Governance of Low-Carbon Futures	[61]	International Journal of Urban and Regional Research	2014	165	0
Constructing sustainable tourism development: The 2030 agenda and the managerial ecology of sustainable tourism	[62]	Journal of Sustainable Tourism	2019	160	0
The Glue and the Pieces – Entrepreneurship and Innovation in Small-firm Networks	[63]	Journal of Business Venturing	1994	144	0
Competitiveness of Small Farms and Innovative Food Supply Chains; The role of Food Hubs in Creating Sustainable Regional and Local Food Systems	[66]	Sustainability	2016	128	0
The Fourth Industrial Revolution (Industry 4.0): A Social Innovation Perspective	[67]	Technology Innovation Management Review	2017	121	0
Measuring Entrepreneurship over time	[68]	Journal of Business Venturing	1995	116	0
Governing public-private partnerships for sustainability: An analysis of procurement and governance practices of PPP infrastructure projects	[69]	International Journal of Project Management	2017	108	0
Impacts of urban living labs on sustainability transitions: mechanisms and strategies for systemic change through experimentation	[70]	European Planning Studies	2019	90	0
The impacts of higher education institutions on sustainable development: a review and conceptualization	[64]	International Journal of Sustainability in Higher Education	2019	83	1
Putting sustainable supply chain management into base of the pyramid research	[65]	Supply Chain Management – An International Journal	2015	70	3

In terms of key contributions to the current SLR [61], provide valuable insights into urban sustainability, emphasizing the importance of urban laboratories in promoting a low-carbon future. The significance of this type of living laboratory is emphasized, particularly regarding testing and approaches in a controlled environment, involving various stakeholders and communities in the policy process, and generating learning and innovation.

The study developed by Ref. [62], provides an intriguing perspective of the role of sustainable tourism in achieving the United Nations 2030 Agenda for Sustainable Development. Similarly, in Ref. [62] it is recognized the need for a “managerial ecology” approach to sustainable tourism development. This entails a holistic and integrated management approach, also linked to triple bottom line sustainability, which integrates economic, environmental, and social dimensions. Besides what has already been stated, the author believes that greater integration and collaboration among all stakeholders, the development of sustainable policies and regulations, and the implementation of sustainable practices at the local level are all important.

The role of networks in promoting entrepreneurship and innovation in small businesses is evaluated by Ref. [63]. In the same study [63], it is revealed, firstly, that when companies are closely connected with other firms and organizations, they are more likely to innovate and thus succeed. Secondly, it is stated that these networks are a “glue” that allows companies to gain access to critical resources such as knowledge, skills, and funding, as well as a source of social support and legitimacy. Thirdly, networks and collaborative approaches are positioned as an accelerator for entrepreneurship and innovation.

The three previously reviewed top 3 cited articles make valuable contributions to the current SLR, particularly in interrelated theoretical approaches concerning strategic alliances, sustainability, innovation, and entrepreneurship, which are fuelled by collaborative efforts.

However, it is worth noting that when we examine the number of links pertaining to the top 10 articles, we confirm that there are no links between the top 3 and the remaining articles in the database. In fact, only two of the top ten articles in Table 3 have links: [64,65]. In Ref. [64], it is conceptualized and identified the direct and indirect impacts of higher education on sustainable development.

In turn [65], examines how sustainable supply chain management arguments are linked to the poorest populations, known as the “base of the pyramid,” demonstrating, among other things, the need to foster joint development and innovation in order to drive the global sustainable development agenda.

**Fig. 5:**
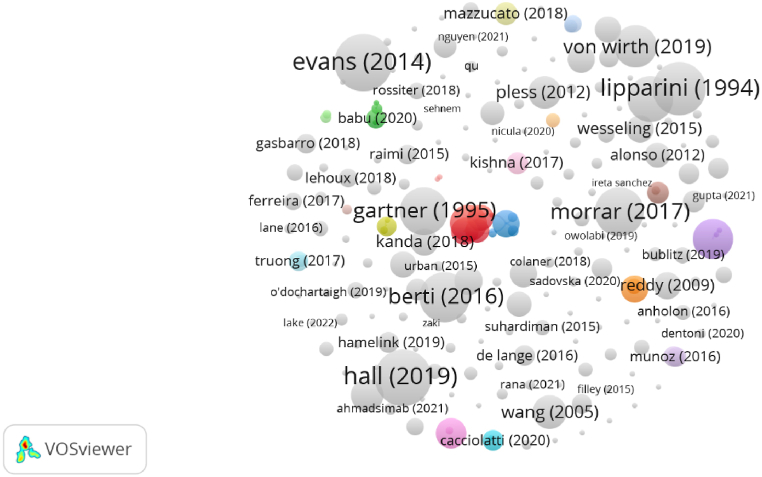
Citation document links (Source: own elaboration using VOS viewer software).

The fact that both articles are related to most aspects of the topics targeted in the SLR can justify the linkage with other articles in the database. Fig. 5 shows that the density of linkages among the articles is low. Nonetheless, it is worth noting that all the articles represented by grey balls have no links in the database, while those represented by coloured balls have links.

**Fig. 6:**
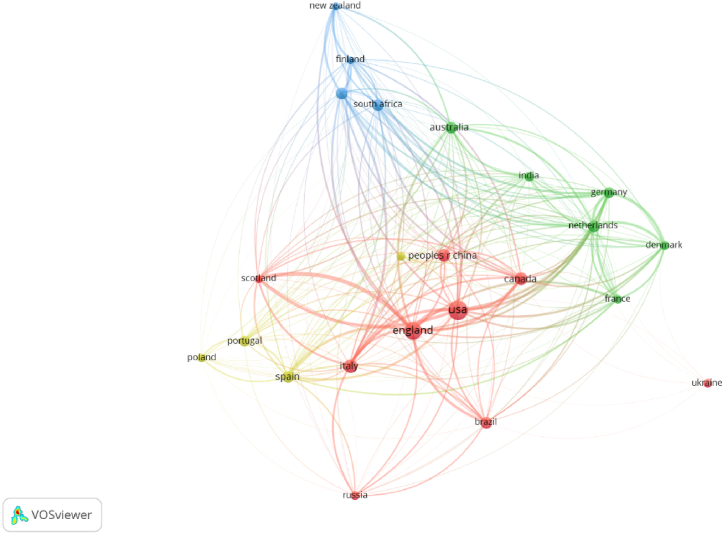
Location Connection Network (Source: own elaboration using VOS viewer software).

When analysing the top locations regarding the publications included in this systematic approach, the United States of America has most publications, with a total of 35, followed by England with 29, as seen in Table 4. Fig. 6 indicates the strengths of the connection networks between locations.

**Table 4:** Locations with most publications.

Location	Number of Publications
USA	35
ENGLAND	29
ITALY	17
CANADA	14
CHINA	13
AUSTRALIA	12
SPAIN	12
BRAZIL	11
NETHERLANDS	11
SOUTH AFRICA	11

### 4.2 Three-pronged approach

#### 4.2.1 Topic mapping analysis

Concerning the procedures for preparing the topic mapping analysis, extraction and construction of the database from WoS enabled us to collect a dataset that did not require the elimination or merging of similar keywords. However, the option of revising and standardizing the entries to English (UK) was selected, with the removal of plural words, as well as abbreviations and JEL code, being protected. Given the novelty of the topic of this SLR, no threshold of occurrences was established to ensure the inclusion of a total number of articles that would allow for the bibliometric analysis and subsequent presentation of the framework research.

**Fig. 7:**
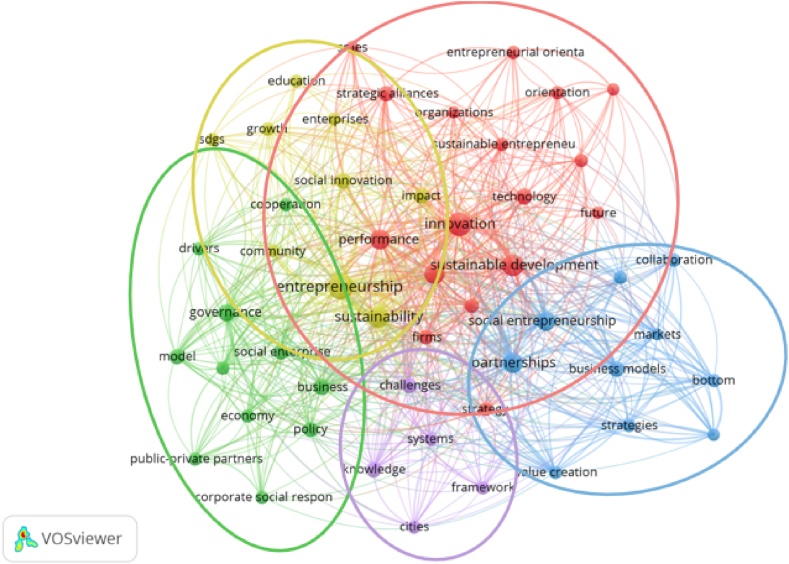
Word Co-Occurrence (Source: own elaboration using VOS viewer software).

Fig. 7 shows the co-word occurrence regarding the publication title, keywords and abstract. The most relevant subjects identified are ‘entrepreneurship’ (59 occurrences), ‘innovation’ (44), ‘sustainability’ (36), ‘sustainable development’ (36), ‘partnerships’ (27), ‘performance’ (25), ‘management’ (25), ‘social entrepreneurship’ (21), ‘governance’ (17), and ‘technology’ (13).

This analysis took into consideration the title, abstract and keywords of all 207 articles, using VOSviewer software. A binary counting method was used in order to identify whether the reference word appears in the document. Taking as reference Fig. 7, we can identify 5 clusters, based on the relationship of the different reference words and their association. The first cluster (yellow) is composed of 9 terms or words, with the most relevant being ‘sustainability’, ‘entrepreneurship’, ‘impact’, ‘community’ and ‘social innovation’. This cluster was named: Entrepreneurship's impact on community sustainability and social innovation.

The second cluster (red) is composed of 13 terms, including ‘sustainable development’, ‘innovation’, ‘performance’, ‘strategic alliances’ and ‘sustainable entrepreneurship’. This cluster was named: Strategic alliances for sustainable development, innovation and performance.

The third cluster (blue) is composed of 8 terms, with the most relevant being social ‘entrepreneurship’, ‘partnerships’, ‘value creation’, and ‘business models’. This cluster was named: Value creation through partnerships in social entrepreneurship.

The fourth cluster (purple) is composed of 5 terms, the most prevalent being ‘challenges’, ‘knowledge’, ‘framework’ and ‘cities’. This cluster was named: Challenges for knowledge-based sustainable cities.

The fifth cluster (green) is composed of 10 terms, the most relevant being ‘social enterprise’, ‘cooperation’, ‘business’, ‘governance’ and ‘policy’. This cluster was named: Collaboration between businesses and social enterprises.

Cluster 1: Entrepreneurship's impact on community sustainability and social innovation.

According to Ref. [71], social innovation is related to all the practices that aim to solve social needs and improve education, working conditions and health access for the population, in brief, improving community life conditions. Social innovation can also be linked to enhancing future solutions for the population, responding to each community's needs. To tackle those societal needs, various interested parties involved in the community are called to define what to do, and how to do it. This relationship should be fostered through exchange and collaboration among the various stakeholders, connecting national, regional and community political leaders with other relevant stakeholders, such as companies and HEIs [72]. In order to have long-lasting outcomes [73], state that in social innovation there must be participation by citizens, not only as users, but also as ‘consultants’, as co-creators of the decision process. One of the main sources of social innovation is participatory budgeting, allowing the transformation of local governance and assuring citizens' inclusion [74]. Social innovation is society's attempt to correct certain actions, complementing the public sector. It intends to be a better way to support the community besides subsidies or through governments' social enterprises [75]. Considering HEIs' third mission, and their opening up to the surrounding society, engaging in non-academic activities, HEIs can alter the entrepreneurial environment in communities by bringing their research into possible new companies, training new employees focusing on the regions' workforce necessities, and also by stimulating through business incubators. This would lead to families settling in these regions, the creation of new companies and new jobs [25,76].

#### 4.2.2 Cluster 2: strategic alliances for sustainable development, innovation, and performance

The strategic alliances are defined in Ref. [4], as a cooperation between parties that uses resources from both sides. These cooperative interfirm arrangements aim to reach common goals and can take several forms, such as joint ventures, direct equity investments, or joint market arrangements [14]. According to Ref. [77], cross-sectoral strategic alliances are mechanisms that bring together competencies and knowledge for shared value purposes, providing the resources of all for better answers to the challenges of sustainable development, such as those related to the environment. Alliances can also contribute to treatment for major diseases, sustainable finance, and sustainable development, which can lead to new sustainable companies and jobs. There is integrated sustainable development as a driving force for all kinds of stakeholders involved. The strategic alliances provide resources and capacities that organizations could not reach individually, allowing the development of three dimensions: economic, social, and environmental [78].

In this line of thought, and considering the SDGs of the United Nations 2030 [41], it is important to point out that the final goal (SDG 17) is fully directed to developing greater connections and partnerships for sustainable development, so that governments can work towards strong, fully operational cooperation to implement the SDGs. For this, the partnerships should be at a global, regional, national, or subnational level, and should aim to share knowledge, technology and expertise for better implementation of the SDGs in the regions.

#### 4.2.3 Cluster 3: value creation through partnerships in social entrepreneurship

The concept of value creation has been evolving over time, and nowadays with the integration of clients and customers as co-creators of value, value creation takes place through an interactive process of utilization [79]. This was the main idea when service-dominant logic started to consider clients’ perspectives in value creation, taking into account all the values that are brought, becoming co-creators [[80], [81], [82]]. This connection in an environment that spurs the interaction and participation of both parties improves value creation [83]. But are partnerships in social entrepreneurship vehicles for greater value creation? Can these partnerships be important for value creation in social entrepreneurship?

As stated in Ref. [84], the social entrepreneurship can be related to a set of behaviours that do not fit all kinds of social sector leaders, in the same way that not all business leaders are entrepreneurs. In the same vein, the social entrepreneur as being able to go further in creating social value, exploring opportunities that will impact on the community, society or the world [84]. For this, the social entrepreneur must recognize and take advantage of opportunities that arise, and there must be a continuous process of innovation and learning, in order to act and engage in entrepreneurial actions, not worrying about resources that are generally scarce. According to Ref. [85], these characteristics are not enough for the future economic performance of social enterprises, and so these severe resource constraints are one of the main incentives for a partnership. This interest in collaborative value creation, as stated by Refs. [86,87], is a facilitation process, where both parties (whether large or small corporations or enterprises), despite having doubts about one another regarding the social partnership, enable social partnership for co-creation and provide the resources each party needs.

Cluster 4: Challenges for knowledge-based sustainable cities.

The HEIs and regions align to build partnerships, contributing to developing knowledge-based industries and human capital, which leads to new companies and jobs, and greater revenue for the locals [88]. Moreover, the HEIs are moving away from their previous introspective positioning, which was mainly focused on being sources of knowledge, to develop high-tech innovation and new knowledge for industry [89]. HEIs are now seen in a broader perspective, as a stakeholder of regional and national influence, decisively affecting the social fabric of their regions. This is in line with the statement in Ref. [90], pointing out that the knowledge-based economy places HEIs as central for regional development, shifting from its traditional role as a static player to a more proactive role.

According to Ref. [91], we should consider knowledge-based development (KBD), since it is from knowledge that regional development takes place, and so it must be managed more effectively. That implication was already seen by Ref. [92], and applied to cities as a stepping-stone to sustainable development by using their own knowledge resources for better development. The continuous growth and complexity of cities will create new adversities, as well as new opportunities for a more sustainable framework for development [93].

Following [94], the idea of a Smart City Service System (SCSS) implies the sharing of data, information and opinions related to people, organizations, providers, users and entities, who will co-create value for the city and thus improve services through exchanging information with service usage. In this vision, cities could have tailor-made services, optimizing for instance waste management or public transport. Notwithstanding, as outlined in Ref. [95], there are some difficulties in gathering data, due to the complexity of elements in knowledge management, and associated with the high costs of gathering and treating all the data. As indicated in Ref. [96], there is a difficulty for cities changing from industrial to knowledge-based, since nowadays both paradigms coexist, and this together with all possible changes (and difficulties traditionally faced with change) are major barriers to a rapid shift.

#### 4.2.4 Cluster 5: collaboration between businesses and social enterprises

The European Commission has been a key-actor in highlighting social enterprises throughout Europe, raising awareness of their role in facing up to environmental changes and social difficulties [97]. The importance of social enterprises is also linked to the greatest challenges foreseen for the 21st century by the World Economic Forum regarding social, economic and environmental development. Of all the needs connected with sustainability and human development, only a small part can be fulfilled by governments or international organizations [98].

According to Ref. [99], social enterprises are called to respond to typical social, cultural and environmental problems, when governments or companies are unable to respond (due to scarcity of funds or the low profit to be made). There is sometimes the need to engage in alliances between business and the social aspect in order to deal with complex problems in society, which could not be solved individually. As concluded by Ref. [100], in their study on the reasons for business and social enterprise cooperation, when applying resources, the cooperation between business companies and social enterprises co-generates social value, with the companies providing funds, and social enterprises providing knowledge, expertise, organizational infrastructure and established social networks.

Despite the importance of alliances or cooperation between corporate and social entrepreneurs, the subject of corporate social entrepreneurship is yet to be tackled by HEIs [101]. This is especially due to the lack of finance sources which could foster the shared value originating from University strategic alliances, including government bodies and different types of stakeholders.

#### 4.2.5 Co-citation analysis

First, a co-citation analysis of co-cited authors was performed for deeper study of the roadmap followed in the field. To avoid overcrowding, only papers with at least 20 citations were used, and only the “first author” name was used.

**Fig. 8:**
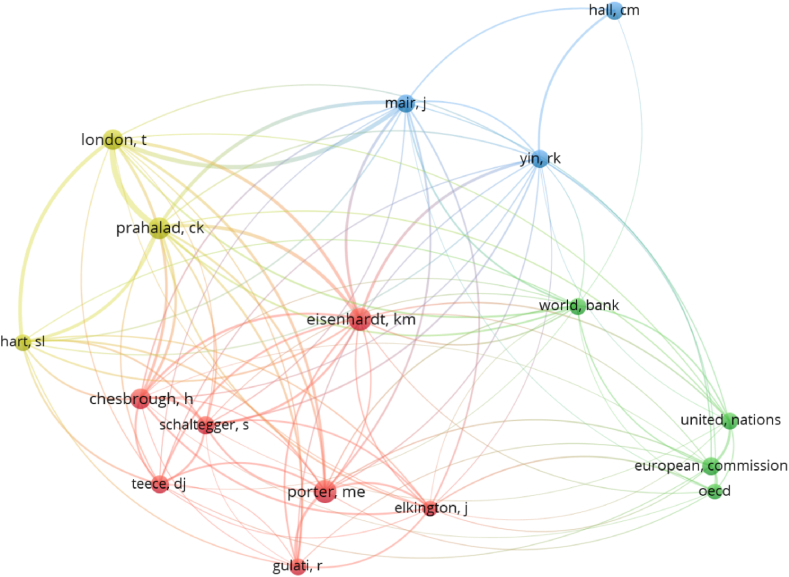
Author co-citation clusters in HEIs' strategic alliances and sustainable entrepreneurship (1994–2022) (by first author; citation ≥20) (Source: own elaboration using VOS viewer software).

Fig. 8 shows co-citations from 1994 to 2022, with larger nodes indicating more citations. The findings reveal four major clusters, namely “Strategic alliance - innovation” (Chesbrough, Einsenhardt, Elkington, Gulati, Porter, Schaltegger, and Teece), “Entrepreneurship - Sustainable development” (European Commission, OECD, United Nations, and World Bank), “Sustainable innovation - Sustainable entrepreneurship” (Hall, Mair, and Yin), “Base of the Pyramid-Co-creation” (Hart, London and Prahalad). These co-citation clusters are consistent with the previous topic mapping clusters, revealing a linkage originating from strategic alliances, entrepreneurship, and innovation frameworks aimed at new exploration pathways devoted to sustainable development, social entrepreneurship and co-creation.

**Fig. 9:**
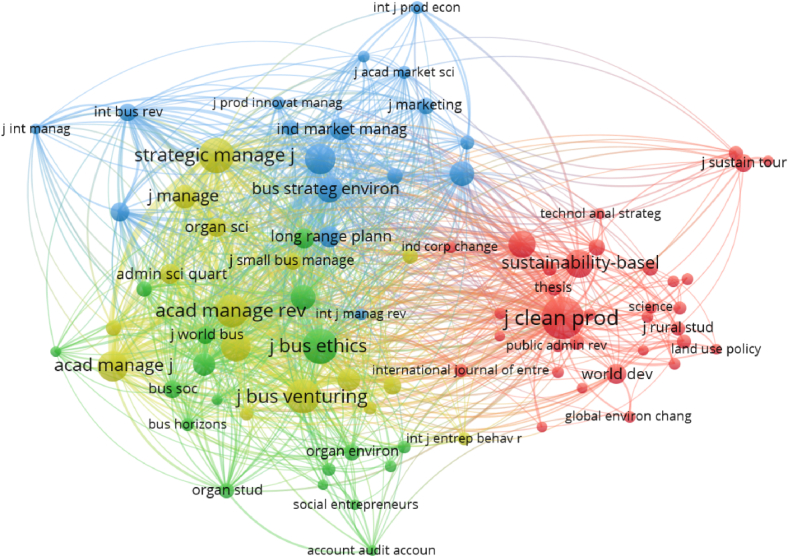
Journal-based co-citation clusters in HEIs' strategic alliances and sustainable entrepreneurship (1994–2022), by journal sources, with citations ≥20) (Source: own elaboration using VOS viewer software).

Second, to deepen the insights, a co-citation analysis at the journal level was carried out, using journal sources with at least 20 citations for the entire period. The results are shown in Fig. 9 and represent a diverse and complex set of journal-based co-citation clusters, including: “Natural resources - Sustainable development” (Journal of Cleaner Production, Sustainability, Research Policy, World Development, and Energy Policy), “Base of the pyramid - Social business” (Journal of Business Ethics, Harvard Business Review, Business and Society, Journal of Management Studies, and Journal of World Business), “Co-creation - Sustainable innovation” (Journal of Business Research, Technological Forecasting and Social Change, Business Strategy and the Environment, Long Range Planning, and Industrial Marketing Management) and “Social Entrepreneurship - Partnerships for Sustainability” (Journal of Business Venturing, Academy of Management Review, Academy of Management Journal, Strategic Management Journal, Entrepreneurship, Theory and Practice). The trends identified in the co-citations at the journal level corresponded to the clusters identified in the previous co-occurrence analysis that yielded the topic mapping.

#### 4.2.6 Overlay analysis of new and hot topics

**Fig. 10:**
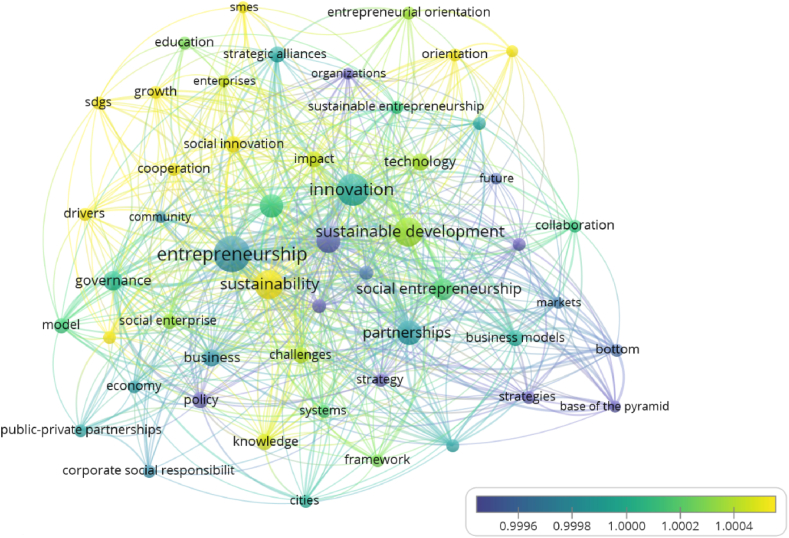
Overlay map of “new topics” in HEIs' strategic alliances and sustainable entrepreneurship (1994–2022) (Source: own elaboration using VOS viewer software).

First, the terms in the topic clusters were matched with the corresponding publication year, with the most recent topics visualized in a colour ranging from green (relatively new) to yellow (the newest), and the oldest in a colour ranging from blue (relatively old) to purple (the oldest), using a normalized scale. This option provides a colour-based visualization of newer and older publications. Fig. 10 depicts the trends in articles on new topics, such as the newest, which include SDGs, growth, orientation, drivers, sustainability, social innovation, and knowledge (clusters 1, 2, and 5), and the relatively new, which include entrepreneurial orientation, education, sustainable development, social enterprise, impact, and technology (clusters 3 and 4).

**Fig. 11:**
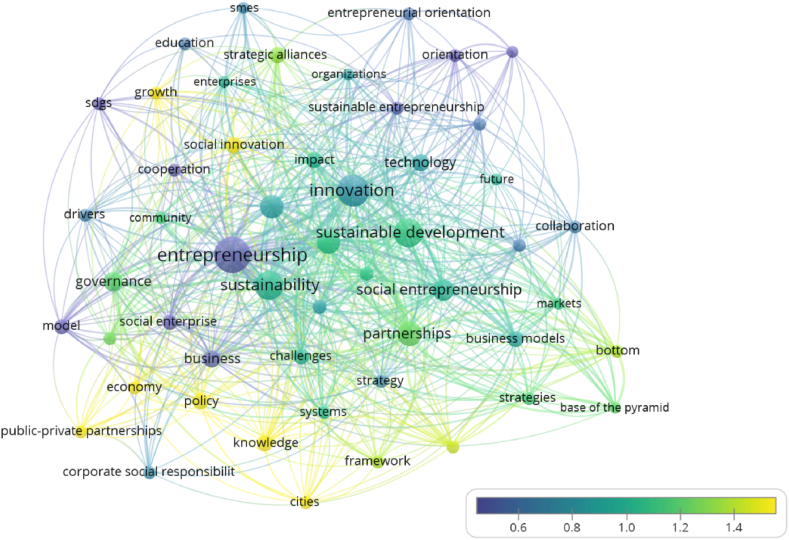
Overlay map of “hot topics” in HEIs' strategic alliances and sustainable entrepreneurship (1994–2022). (Source: own elaboration using VOS viewer software).

Next, the terms in the topic clusters were matched with the citation score of the publication where the terms were found, using average normalized citations, to generate the “hot topics” via the citation-based overlay visualization. These normalized citation scores were averaged using a range of colours from purple (coldest) to blue (relatively cold), green (relatively hot), and yellow (hottest publications). As a result, terms with the highest citation impact were highlighted in yellow, and terms with the lowest citation impact were highlighted in purple, resulting in a graphical representation of ‘hot’ versus ‘cold’ publications, as shown in Fig. 11. Social innovation, growth and knowledge (clusters 1, 2, and 5), public-private partnerships (cluster 3), and cities, economy and policy are among the “hot topics” (cluster 4).

## 5 Discussion and research framework proposal

According to Ref. [55], by implementing a three-pronged approach, it was possible, in a first step, to identify five topic clusters based on a word co-occurrence analysis, namely: (1) Entrepreneurship's impact on community sustainability and social innovation; (2) Strategic alliances for sustainable development, innovation and performance; (3) Value creation through partnerships in social entrepreneurship; (4) Challenges for knowledge-based sustainable cities; and (5) Collaboration between businesses and social enterprises.

In a second step, the co-citation analysis validates the previously identified topic mapping clusters by connecting the classical theoretical approaches based on entrepreneurship and innovation with the established literature stream devoted to strategic alliances. Furthermore, it reveals new pathways associated with recent extensions concerning sustainable development, co-creation, sustainable entrepreneurship and social innovation.

In a third step, the overlay visualization advances the still scarce knowledge about the citations' impact, highlighting as hot topics: social innovation linked to knowledge and growth; public-private partnerships as a joint cooperation mechanism; and the innovative nature of economic policy focused on cities, where knowledge, co-creation, sustainable entrepreneurship and social innovation are critical levers to ensure sustainable development.

In the last decade, social and environmental practices and issues have given entrepreneurs several new opportunities, giving rise to sustainable or environmental entrepreneurs, as well as social entrepreneurs [102]. The appearance of sustainable or environmental entrepreneurs is not only linked to traditional ways of ensuring wealth, but also to fighting for better environmental and sustainable conditions for the present and coming generations [103]. This is aligned with the vision presented by Ref. [10], stating that sustainable entrepreneurship and entrepreneurs are connected to sustainable innovations directed to consumers, also allowing greater benefits for surrounding communities and society.

This development of sustainable entrepreneurship is in line with the direction of the European Commission, which wanted to include HEIs in educating future “green entrepreneurs” and thus adopting the mind-set of sustainable entrepreneurship, through building opportunities and cooperation with other HEIs, as well as with business [104]. For its turn, the entrepreneurial HEI should be interactive but also an independent player that can also engage in alliances, partnerships or cooperation with industry and government, in order to benefit communities [105]. HEIs are a particular player in developing a special mission, since they must be considered as an engine of growth [105,106]. This engine should spur a responsible change in society, based on sustainability, crossing economic, environmental and social layers. This should be positioned at the heart of the “Third Mission of HEIs”, impacting greatly on the surrounding regions and on the people living there [107].

Joint cooperation and alliances between HEIs, but also between HEIs and other stakeholders, is in line with the European Commission's view of sustainable entrepreneurship. Moreover, in Ref. [108] it is advocated that HEIs are major players and contribute greatly to improving sustainability worldwide, through their missions of education and research, and because they can collaborate with several stakeholders [109]. This agrees with the statements provided by Ref. [28], who outlined the importance of the relationships between HEIs and public and private organizations as potential sources of innovation and for economic, environmental and social development, which is vital for the so-called ‘knowledge societies’, or ‘knowledge-based economies’. As stated by Ref. [110], the services HEIs provide to society through the ‘third mission’ are an exploration of knowledge and expertise, as well as its applicability by the HEIs, or other entities, to benefit the community. Thus, Knowledge-Based Development will connect what is being developed in HEIs, in order to transfer that knowledge to society, thus spurring social entrepreneurship, sustainable entrepreneurship, and social innovation [8,92,111].

**Fig. 12:**
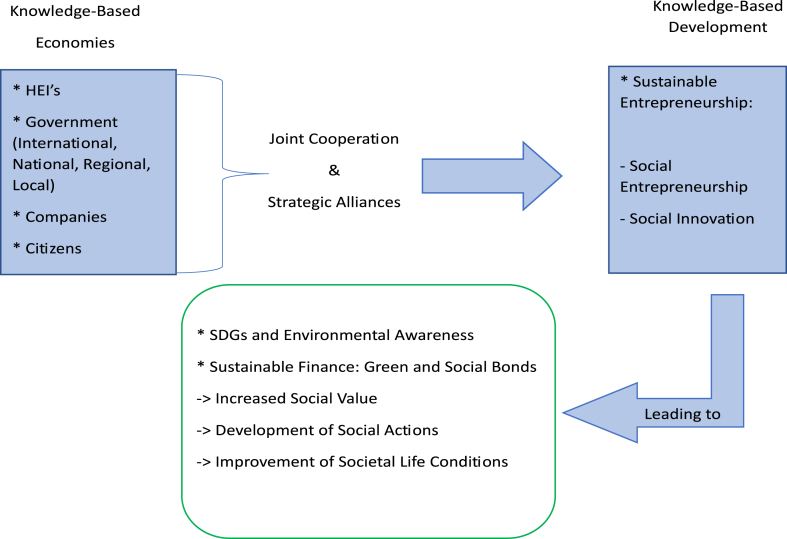
Joint Cooperation and University Strategic Alliances in Sustainable Entrepreneurship: Proposal of a research framework (Source: own elaboration).

From the results obtained through this SLR, with the two-fold purpose of mapping the state-of-art and preparing future endeavours, a research framework proposal regarding joint cooperation and strategic alliances for sustainable entrepreneurship was designed, as presented in Fig. 12 below.

In order to understand how the main actors in knowledge-based economies (HEIs, Industry, Government, and Citizens) can contribute to knowledge-based development, there is a need to integrate the five topic clusters. These cover joint cooperation and universities' strategic alliances, in the form of University-Industry relations, University-University relations, sustainable entrepreneurship, and universities' social impact. It is also important to ensure alternative fund-raising mechanisms targeted at social and environmental innovation activities, to foster universities’ strategic positioning, in terms of achieving then SDGs and environmental awareness.

This holistic research framework integrates the still unexplored set of relationships made through joint cooperation and strategic alliances between the major stakeholders of Knowledge-Based Economies (HEIs, Government, and Companies), which often lead to Knowledge-Based Development, based on Sustainable Entrepreneurship. This development and innovative efforts are often connected to SDGs and Environmental Awareness and to the availability of sustainable finance sources, for the whole community and society that is both directly and indirectly connected with HEIs. Knowledge-based development will also lead to increased social value and the development of social actions, which can lead to improved living conditions in society and to reinforcement of the shared value based on exploiting alternative sustainable finance sources, such as green and social bonds targeted to achieving SDGs.

It is important to point out the importance of HEIs for the surrounding community, as well as the connections and relationships they can develop with stakeholders, including government, companies and citizens. This importance is connected not only with the greater development of HEIs and the connected stakeholders, but also with development of the surrounding community and improved social and environmental conditions.

## 6 Conclusion

This SLR encompassed 207 articles and intended to provide a state-of-the-art overview of HEIs' joint cooperation and strategic alliances targeted to sustainable entrepreneurship. In this summary, taking as a cornerstone the relationship between HEIs' strategic alliances and sustainable entrepreneurship, in relation to the first research question (Q1), this SLR mapped five topic clusters: (1) Entrepreneurship's impact on community sustainability and social innovation; (2) Strategic alliances for sustainable development, innovation, and performance; (3) Value creation through partnerships in social entrepreneurship; (4) Challenges for knowledge-based sustainable cities; and (5) Collaboration between businesses and social enterprises.

Addressing the second research question (Q2), integration of the five topic clusters with a sustainable development meaning allowed the design of a holistic framework, joining the main actors of knowledge-based economies (HEIs, Government, Companies and Citizens), committed to cooperation and strategic alliances, to ensure knowledge-based development, anchored on sustainable entrepreneurship, social entrepreneurship and social innovation. This complex type of strategic alliance can facilitate the emergence of entrepreneurial ecosystems, spurring sustainable entrepreneurship initiatives, and achievement of the targets associated with the SDGs, as well as the necessary fundraising through green finance sources, such as green bonds and social bonds. This holistic approach will lead to increased social value, development of social actions, and improved living conditions, based on HEIs’ environmentally friendly and socially responsible actions.

Furthermore, the three-pronged approach enabled the development of solid, rigorous argumentation supported by a three-step analysis, which included co-word occurrence, co-citation analysis and overlay visualization.

In the first step, co-citation clusters guided our bibliometric analysis from established perspectives devoted to strategic alliances, entrepreneurship and innovation to emergent visions linked by a common theoretical argumentation in favour of sustainable development, acting as an increasingly accepted “glue” with ongoing advances related to sustainable entrepreneurship, social entrepreneurship, and co-creation being increasingly prominent.

In a subsequent step, analysis of a diverse and complex set of journal-based co-citation clusters revealed that the pathways leading to presentation of a new holistic framework research devoted to: Joint Cooperation and University Strategic Alliances in Sustainable Entrepreneurship, are sourced in perspectives originating from the nexus: Natural resources - Sustainable Development, evolving to different but interconnected exploration pathways including: Base of the Pyramid – Social business; Co-creation - Sustainable innovation; and Social Entrepreneurship - Partnerships for Sustainability. These findings support the importance of fostering joint cooperation among HEIs, government, businesses and citizens, all sharing knowledge, co-creating for the common good, and putting social innovation at the heart of joint cooperation.

In a third step, using normalized citation scores enabled the overlay visualization, revealing that social innovation, growth and knowledge are hot topics that merit additional research efforts and policy action. Smart tools aimed at collaboration, such as public-private partnerships, are critical for launching a new generation of sustainable policies with positive economic, environmental and social impacts. A new city concept is also required, reinforcing the links between urban and rural dimensions while employing sustainable and nature-based solutions.

To summarize the findings from the three-pronged approach, the success of the proposed joint cooperation framework and agenda is dependent on the ability to open up the knowledge existing in HEIs to society, using civic co-creation approaches, with the goal of fostering sustainable entrepreneurship and social innovation, in order to ensure sustainable development.

HEIs are currently an extremely important stakeholder for communities, being a focus for knowledge, research and development of ideas. By making connections with other HEIs and stakeholders, further developments are triggered in knowledge societies, which can lead to impactful social and environmental change. It is worth emphasizing the importance of providing a new research framework, including both knowledge-based economies and knowledge-based development, leading to increased social value and environmental awareness based on exploiting sustainable finance sources, such as green and social bonds. This has the potential to deepen the shared value orientation and responsibility of public bodies, investors and citizens.

This SLR is not without limitations, since only scientific articles were analysed, without considering other references, such as books, chapters or reports. In addition, the clusters were defined through the authors' lens, so their dimension and analysis could be different if viewed from other lenses and perspectives. The literature was collected from only one source, Clarivate's Web of Science (WoS) database, which despite being the most utilized database for this kind of study is certainly not the only one.

During this research, several gaps were identified, mostly related to differentiating the types of cooperation and alliances HEIs engage in. Conversely, despite the existence of some articles on HEI-HEI alliances and others on HEI-Company alliances, there is no differentiation regarding the outputs and inputs for each partner. Few articles focus on the sustainable entrepreneurship arising from these types of alliances or cooperation. Therefore, there is no deep understanding of the impact of HEI joint cooperation and alliances on sustainable entrepreneurship and this gap could be further analysed in future research endeavours.

It could also be important to engage in case studies to deepen knowledge about joint cooperation and strategic alliances between HEIs (and between HEIs and other stakeholders) when they ended badly, or at least did not develop or improve sustainable entrepreneurship based on social entrepreneurship and social innovation. With this type of analysis, we could better understand how and why these alliances fail regarding sustainable entrepreneurship. Lastly, it could be interesting to deepen the relations between HEIs and the different stakeholders concerning co-creation, co-sharing, co-financing and open innovation, using an Environmental, Social and Governance (ESG) approach, aiming to assess the impacts of these relations on the surrounding communities, mostly in terms of quality of life and sustainability.

This development and innovative efforts are often connected to a greater focus on SDGs and Environmental Awareness and to the availability of sustainable finance sources, for the whole community and society that is both directly and indirectly connected with HEIs. Knowledge-based development will also lead to an increased social value and the development of social actions, which can lead to improved living conditions, and to reinforcing the shared value based on exploiting alternative sustainable finance sources, such as green and social bonds targeted to achieving SDGs.

Environmental issues, living conditions, sustainability and SDGs are becoming top priorities for the majority of governments (international, national, regional or local), HEIs, companies and citizens. Therefore, all knowledge-based analyses of the relationship between strategic alliances and sustainable entrepreneurship are of critical importance and arouse the interest not only of the stakeholders identified, but also of future entrepreneurs dealing with societal challenges. The analysis conducted aims to be a useful research framework that can be used to expand the still limited knowledge on the topic of sustainable entrepreneurship resulting from strategic alliances with HEIs, which have increasing responsibility as knowledge cornerstones in fostering a sustainable future for the communities they are part of.

## Funding

The Portuguese Foundation for Science and Technology (Grants and NECE- UIDB/04,630/2020) provided financial support for this study.

## Author contribution statement

All authors listed have significantly contributed to the development and the writing of this article.

## Data availability statement

Data included in article/supplementary material/referenced in article.

## Additional information

Supplementary content related to this article has been published online at [URL].

## Appendix A Supplementary data

Supporting Information.docx Supporting Information.docx

## Footnotes

1 For further details about the European University Alliance, please consult: https://univ-unita.eu/Sites/.

2 For further details, please consult the EcoAction platform: https://eit-hei.eu/projects/ecoaction/.
